# CauloBrowser: A systems biology resource for *Caulobacter crescentus*

**DOI:** 10.1093/nar/gkv1050

**Published:** 2015-10-17

**Authors:** Keren Lasker, Jared M. Schrader, Yifei Men, Tyler Marshik, David L. Dill, Harley H. McAdams, Lucy Shapiro

**Affiliations:** 1 Department of Developmental Biology, Stanford University, Stanford, CA 94305, USA; 2 Department of Computer Science, Stanford University, Stanford, CA 94305, USA

## Abstract

*Caulobacter crescentus* is a premier model organism for studying the molecular basis of cellular asymmetry. The *Caulobacter* community has generated a wealth of high-throughput spatiotemporal databases including data from gene expression profiling experiments (microarrays, RNA-seq, ChIP-seq, ribosome profiling, LC-ms proteomics), gene essentiality studies (Tn-seq), genome wide protein localization studies, and global chromosome methylation analyses (SMRT sequencing). A major challenge involves the integration of these diverse data sets into one comprehensive community resource. To address this need, we have generated *CauloBrowser* (www.caulobrowser.org), an online resource for *Caulobacter* studies. This site provides a user-friendly interface for quickly searching genes of interest and downloading genome-wide results. Search results about individual genes are displayed as tables, graphs of time resolved expression profiles, and schematics of protein localization throughout the cell cycle. In addition, the site provides a genome viewer that enables customizable visualization of all published high-throughput genomic data. The depth and diversity of data sets collected by the *Caulobacter* community makes *CauloBrowser* a unique and valuable systems biology resource.

## INTRODUCTION

The bacterium *Caulobacter crescentus* is a valuable model system for cell cycle control in which the transcriptional genetic circuitry is interwoven with the 3D deployment of regulatory and structural proteins (1,2). Each cell division in *Caulobacter* is asymmetric and produces daughter cells with distinct morphology and cell fate. *Caulobacter* cell cycle regulation involves far more than transcriptional networks, and includes differential chromosome methylation, transcription factor activation by spatially-restricted phospho-signaling pathways, temporally and spatially-controlled proteolysis, differential translation, post-translational control of gene expression, subcellular localization of regulatory proteins, and the dynamic topology of the cell; all of which are integrated into a robust regulatory network (3–5).

For the past 20 years, the *Caulobacter* community has taken a systems-based approach to study the molecular mechanisms driving the *Caulobacter* cell cycle. These efforts have generated a wealth of high-throughput time-resolved data gathered at multiple times in the cell cycle; including data from 132 microarray experiments, 13 RNAseq experiments, 8 ribosome profiling experiments, 9 LC-MS mass spectrometry experiments, 7 ChIP-seq experiments revealing hundreds of transcription factor binding sites, a Tn-seq experiment delimiting all essential open reading frames (ORFs) and non-coding elements, 438 global protein subcellular localization experiments, and global chromosomal methylation state throughout the cell cycle.

Given this wealth of data, a major challenge involves the integration of these diverse spatiotemporal data sets into one comprehensive official community resource. To address this need, we have generated *CauloBrowser* (www.caulobrowser.org), an online database that serves as an informatics hub composed of all published experimental data derived from global experiments. This site provides a user-friendly interface for quickly searching genes of interest and downloading genome-wide results. Individual genes are displayed with graphs of time resolved expression profiles for transcription at all promoters and translation at every ORF throughout the *Caulobacter* cell cycle. In addition, the site provides a genome viewer that enables customizable visualization of all available high-throughput genomic data.

## CAULOBROWSER

**Figure 1.**
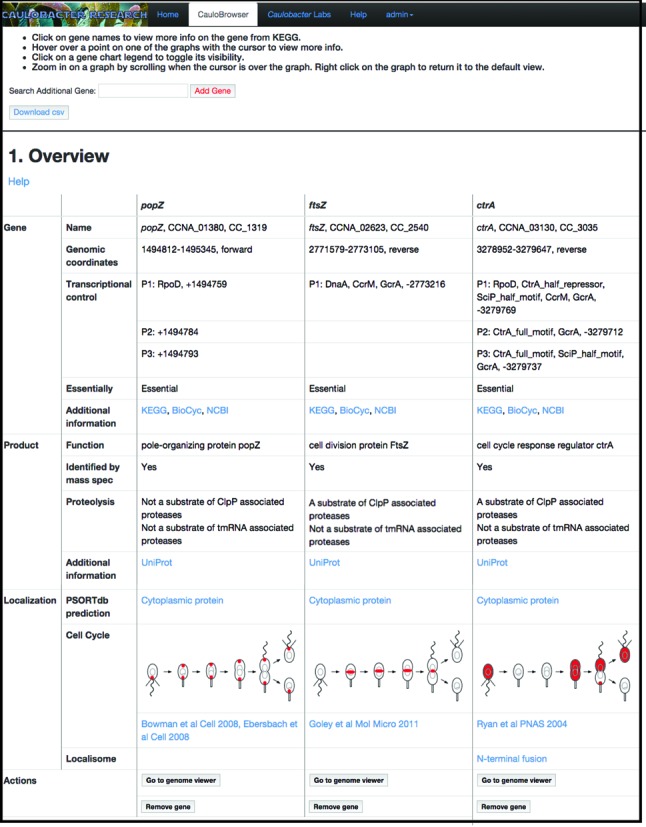
Screenshot of *CauloBrowser* overview section. The figure shows the overview section search results for genes *ctrA, ftsZ and popZ*. The results are organized as a table with a row per data category and a column per gene. Links to central databases such as KEGG, BioCyc, NCBI, and UniProt are colored in light blue. The ‘Actions’ row provides two buttons. ‘Go to genome viewer’ links to our *Caulobacter* genome viewer page zoomed to the region of the corresponding ORF. ‘Remove gene’ removes the gene from the display.

**Figure 2.**
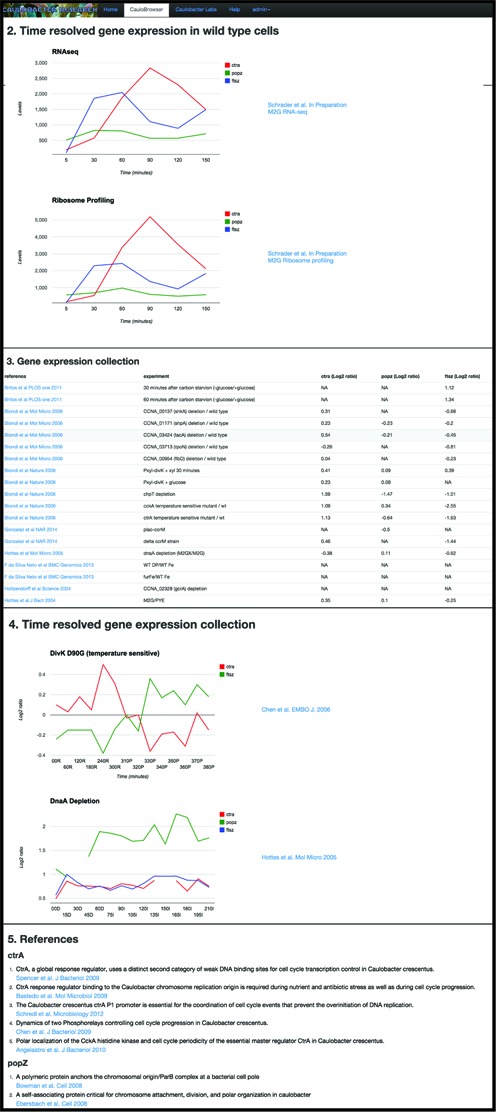
Screenshot of *CauloBrowser sections 2–5*. The figure shows sections 2–5 search results for genes *ctrA* (red)*, ftsZ* (blue), and *popZ* (green). Time resolved expression data (sections 2,4) is visualized using plots of gene expression across the cell cycle in wild type or different growth conditions and mutant background. *Gene expression collection data (section 3)* is organized as a table with a row per experiment and a column per gene. Relevant PubMed references (section 5) are listed with links to corresponding PubMed entries.

*CauloBrowser* start page allows users to search for a gene by its standard name (e.g. *ctr*A) or by its systematic nomenclature (e.g. CCNA_03130 or CC_3035). The user can also search for all genes positioned within a continuous genomic region (e.g. genomic coordinates 1 to 10 000). When specific genes (or a genomic region) have been selected the user is guided to the results page (Figures 1 and 2). The results page is composed of five sections. The first section (‘*Overview*’) provides general information about the function of a gene, the regulation of its expression and the localization pattern of its protein product (if known). The second section (‘*Time resolved gene expression of wild-type cells*’) draws from microarray, RNA-seq, ribosome profiling, and proteomics experiments to provide transcriptional and post-transcriptional plots of gene expression across the cell cycle. The third section (‘*Gene expression collection*’) describes differential expression of genes of interest in a variety of growth conditions (e.g. carbon starvation) and mutant backgrounds (e.g. cell cycle regulatory protein depletions). The fourth section (‘*Time resolved* gene expression *collection*’) provides plots of gene expression across the cell cycle in different growth conditions (e.g. PYE media) and mutant backgrounds. The fifth section (‘*References*’) provides a list of relevant papers. In addition, *CauloBrowser* provides an interactive genome visualization tool that includes tracks for all published genome-scale data.

### OVERVIEW SECTION

The overview section provides a table composed of four categories listing curated information per gene (Figure 1). The first category (‘Gene’) includes the following data. ‘Name’ provides the standard name of the gene, the CCNA locus ID (based on the genome of the NA1000 laboratory strain) (6,7) as well as the CC locus ID (based on the sequence of the closely related CB15 genome) (8). ‘Genomic coordinates’ denotes the protein coding nucleotides of the gene. The ORF coordinates reflect a recent major reannotation (NCBI accession number CP001340.1) (7). ‘Transcriptional control’ lists the experimentally identified transcriptional start sites of the gene as well as any known cell cycle-regulated transcription factor binding sites as determined in (9). ‘Essentiality’ indicates whether the gene was found to be disruptable (i.e. dispensable) or non-disruptable (i.e. essential) for growth in a recent saturating transposon mutagenesis experiment (10). ‘Additional information’ provides corresponding links to the central databases KEGG (11), BioCyc (12) and NCBI (13).

The second category (‘*Product*’) includes the following data. ‘*Function*’ describes the function of the gene product based on NCBI prediction. ‘*Identified by mass spec*’ indicates whether the gene product was identified by LC-MS mass spectrometry in normal laboratory growth conditions (7,14). ‘*Proteolysis*’ indicates whether the gene product is a substrate of ClpP associated proteases (15) and whether it is a substrate of tmRNA associated proteases (16). ‘Additional information’ provides a link to the corresponding UniProt entry (17).

The third category (‘*Localization*’) summarizes the community knowledge on the subcellular localization of the protein. First, the protein is classified as either cytosolic, cytoplasmic-membrane-associated, extracellular, outer-membrane-associated or periplasmic based on PSORTdb, a subcellular localization database for Bacteria and Archaea (18). Second, we provide a cartoon of the protein subcellular localization across the cell cycle. If the subcellular localization pattern is known, usually by observing a fluorescent protein fusion at multiple points in the cell cycle, we draw a corresponding cartoon and cite the relevant paper. If the subcellular localization pattern is not known we provide a predicted cartoon. When the protein is part of stable protein complex we use a known localization pattern of a member of the complex if available. In all other cases we use the predicted PSORTdb localization. We also provide links to images showing the subcellular localization of the protein tagged with a fluorescent protein. These images are a stored in the *Caulobacter* localizome repository, a comprehensive database of protein localization images from a genome-scale imaging screen involving N- and C-terminal fluorescent protein fusions (19).

The fourth category (‘*Actions*’) provides two action buttons. Clicking on ‘Go to genome viewer’ links to our dedicated genome data browser (‘*Genome viewer*’ section). Clicking on ‘Remove gene’ removes the gene from the display.

### TIME RESOLVED GENE EXPRESSION IN WILD TYPE CELLS SECTION

This section provides up to eight time resolved expression graphs per gene. The graphs display individual promoter activity (9), RNAseq and DNA microarrays (20–23)(Schrader *et al*. in preparation), ribosome profiling (Schrader *et al*. in preparation), and proteomics data (24) (Figure 2). The data sets displayed in the top three graphs have been collected and analyzed using the new annotation of the *Caulobacter* genome (7). The RNA-seq, tiling arrays, and ribosome profiling data sets have been normalized such that levels can be compared between genes. The *CauloBrowser* user interface is suited to comparing expression data between multiple genes. All genes searched by the user are displayed in color-coding on the same graph making it easy to perform gene-by-gene comparisons. The graphs provide a toggle (by simply clicking on the gene in the legend) to hide and show the selected genes.

### GENE EXPRESSION COLLECTION SECTION

This section lists a collection of 35 microarrays and 4 proteomics experiments from various different growth conditions and/or strains containing mutations (Figure 2). The table reports the log_2_ ratio between the mRNA or protein levels between the indicated conditions. Two of the data sets show proteins identified as substrates to ClpP protease or substrates of the tmRNA ribosomal rescue system.

### TIME RESOLVED GENE EXPRESSION COLLECTION SECTION

This section provides graphs for a collection of additional cell cycle microarrays performed on mutant strains and/or in various different growth conditions. These experiments track mRNA levels at the indicated time points across the cell cycle (Figure 2).

### REFERENCES SECTION

This final section lists per gene of interest relevant papers (Figure 2). The list is gathered from the gene-centered information resource at NCBI (13). For some genes, e.g. *popZ*, the list of paper generated by NCBI is incomplete and missing key papers. In such cases we manually expanded the list of papers displayed in *CauloBrowser*. We are constantly updating the list of relevant papers per gene and invite the community to participate in this effort.

### NON-CODING RNA

The user can search for a non-coding RNA by its systematic nomenclature (e.g. CCNA_R0001 or CC_t01). The results page is composed of five sections as described above. However, many of the result fields are empty either because they are not relevant or because the corresponding data were not collected. The data displayed for a non-coding RNA includes fields in the overview table and time resolved RNAseq data.

### GENOME VIEWER

**Figure 3.**
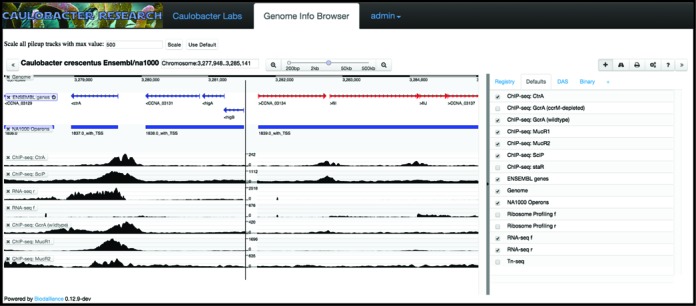
Screenshot of the genome viewer. The figure shows our genomic viewer zoomed to genomic coordinates 3 277 948–3 285 141. Nine tracks are open demonstrating the power of simultaneously visualizing data from different experiments. The top ENSEMBL track shows NA1000 genome annotation with forward genes in red and reverse genes in blue. The second track shows the *Caulobacter* operon structure. Seven additional tracks are shown visualizing multiple Chip-seq and RNS-seq experiments. A list of all available data sets is shown to the right.

*CauloBrowser* contains a genomic data viewer to visualize multiple genome wide data sets (tracks) on the *Caulobacter* chromosome. When loading the genome viewer two default tracks appear. The Genome track displays all ORFs and the NA1000 operons track displays the *Caulobacter* operon structures based upon 5′ Global RACE and RNA-seq data sets (7,9). The user can toggle the visible data sets using the + button. Additional tracks include data gathered from ChIP-seq (25–27), RNA-seq (7,9,20), ribosome profiling (7), and Tn-seq experiments (10) (Figure 3). The user can configure the track display options using the track button, export an image of the data using the printer button, change browsing options using the gear button, and find help using the question mark button. Users can also upload their own data sets using the + button.

## IMPLEMENTATION

*CauloBrowser* (www.caulobrowser.org) web interface was developed using python, HTML, CSS, JavaScript, Google chart tools, and runs on an Apache HTTP server (version 2.4.7) hosted on an Ubuntu Linux server (version 14.04.2 LTS). The *CauloBrowser* database is stored on a MySQL server (version 5.3.43). The Genome viewer was developed based on Dalliance (28).

## OUTLOOK

*CauloBrowser* is a comprehensive resource for the study of *Caulobacter crescentus* cell cycle as a paradigm for asymmetric cell division. *CauloBrowser* allows the user to quickly search genes of interest and browse all published high throughput experiments. We encourage members of the community to take an active role in the development of the portal by providing feedback, suggesting new functionalities as well as additional data sets to visualize. We plan to constantly incorporate new experimental data and maintain the database for at least 5 years. *CauloBrowser* is designed to provide easy access to the multitude of diverse *Caulobacter* data sets. This resource is a valuable tool for the systems level understanding of the spatiotemporal program that controls the *Caulobacter* cell cycle.
